# 3-(2-Meth­oxy­naphthalen-1-yl)-2-benzofuran-1(3*H*)-one

**DOI:** 10.1107/S1600536811026596

**Published:** 2011-07-09

**Authors:** V. Silambarasan, S. Sundaramoorthy, R. Sivasakthikumaran, A. K. Mohanakrishnan, D. Velmurugan

**Affiliations:** a Centre of Advanced Study in Crystallography and Biophysics, University of Madras, Guindy Campus, Chennai 600 025, India; b Department of Organic Chemistry, University of Madras, Guindy Campus, Chennai 600 025, India

## Abstract

The asymmetric unit of the title compound, C_19_H_14_O_3_, contains two crystallographically independent mol­ecules in which the dihedral angles between the naphthalene and benzofuran ring systems are 76.49 (7) and 86.17 (7)°. In the crystal, mol­ecules are linked by inter­molecular C—H⋯O hydrogen-bonding inter­actions into chains running parallel to the *a* axis. In addition, the crystal packing is stabilized by C—H⋯π inter­actions.

## Related literature

For the biological activity of benzofuran compounds, see: Howlett *et al.* (1999 ▶); Aslam *et al.* (2006 ▶); Galal *et al.* (2009 ▶). For natural products with benzofuran rings, see: Akgul & Anil (2003 ▶). For related structures see: Thenmozhi *et al.* (2010 ▶); Valerga *et al.* (2009 ▶). 
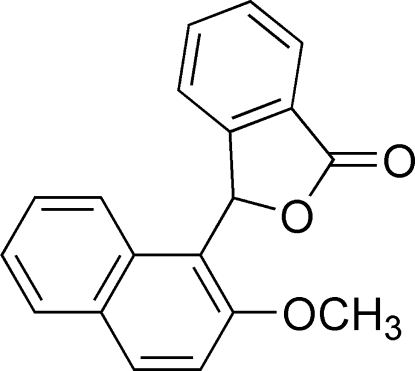


## Experimental

### 

#### Crystal data

C_19_H_14_O_3_
*M* *_r_* = 290.30
Monoclinic, 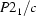

*a* = 13.2572 (5) Å
*b* = 11.8560 (4) Å
*c* = 18.6160 (7) Å
β = 91.657 (2)°
*V* = 2924.79 (18) Å^3^
*Z* = 8
Mo *K*α radiation
μ = 0.09 mm^−1^
*T* = 293 K
0.24 × 0.22 × 0.2 mm

#### Data collection

Bruker SMART APEXII area-detector diffractometer
Absorption correction: multi-scan (*SADABS*; Bruker, 2008 ▶) *T*_min_ = 0.979, *T*_max_ = 0.982
27565 measured reflections
7292 independent reflections
4519 reflections with *I* > 2σ(*I*)
*R*_int_ = 0.027

#### Refinement

*R*[*F* ^2^ > 2σ(*F* ^2^)] = 0.043
*wR*(*F* ^2^) = 0.122
*S* = 1.00
7292 reflections
399 parameters
H-atom parameters constrained
Δρ_max_ = 0.15 e Å^−3^
Δρ_min_ = −0.16 e Å^−3^

### 

Data collection: *APEX2* (Bruker, 2008 ▶); cell refinement: *SAINT* (Bruker, 2008 ▶); data reduction: *SAINT*; program(s) used to solve structure: *SHELXS97* (Sheldrick, 2008 ▶); program(s) used to refine structure: *SHELXL97* (Sheldrick, 2008 ▶); molecular graphics: *ORTEP-3* (Farrugia, 1997 ▶); software used to prepare material for publication: *SHELXL97* and *PLATON* (Spek, 2009 ▶).

## Supplementary Material

Crystal structure: contains datablock(s) global, I. DOI: 10.1107/S1600536811026596/sj5168sup1.cif

Structure factors: contains datablock(s) I. DOI: 10.1107/S1600536811026596/sj5168Isup2.hkl

Supplementary material file. DOI: 10.1107/S1600536811026596/sj5168Isup3.cml

Additional supplementary materials:  crystallographic information; 3D view; checkCIF report

## Figures and Tables

**Table 1:** Hydrogen-bond geometry (Å, °) *Cg*2, *Cg*4 and *Cg*8 are the centroids of C1–C6, C13–C18 and C20–C25 rings, respectively.

*D*—H⋯*A*	*D*—H	H⋯*A*	*D*⋯*A*	*D*—H⋯*A*
C9—H9⋯O6^i^	0.93	2.45	3.238 (2)	142
C14—H14⋯O6^ii^	0.93	2.51	3.435 (2)	175
C12—H12⋯*Cg*4^ii^	0.98	2.93	3.755 (2)	143
C15—H15⋯*Cg*2^iii^	0.93	2.88	3.800 (2)	169
C16—H16⋯*Cg*8^iv^	0.93	2.81	3.553 (2)	137
C30—H30*C*⋯*Cg*8^v^	0.96	2.89	3.687 (2)	141

Symmetry codes: (i) 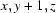; (ii) 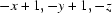; (iii) 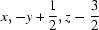; (iv) 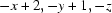; (v) 
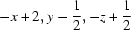
.

**Fig. 1.**
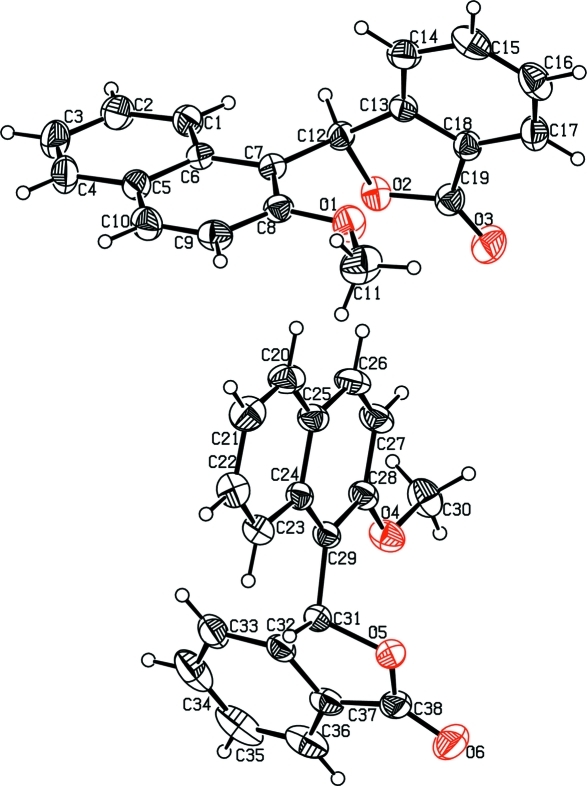
A perspective view of the molecule showing the thermal ellipsoids drawn at the 30% probability level.

**Fig. 2.**
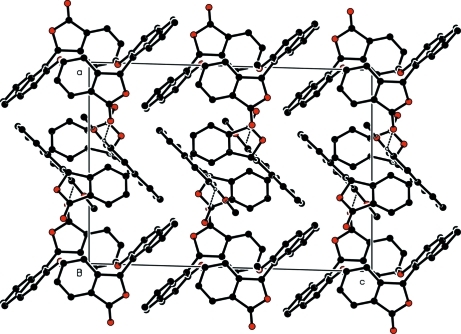
C—H···O interactions (dotted lines) in the crystal structure of the title compound. The crystal packing of the molecules is viewed down the b axis.

## supplementary crystallographic information

### Comment

Molecules containing a benzofuran ring system have attracted considerable interest in view of their biological and pharmacological properties (Howlett *et al.*, 1999; Galal *et al.*, 2009). Compounds with the benzofuran skeleton (Akgul & Anil, 2003) show significant pharmacological activities such as fungicidal (Aslam *et al.*, 2006), antitumor and antiviral activities (Galal *et al.*, 2009). In order to get detailed information such as the geometrical features and the underlying interaction of the crystal structure, an X-ray study of the title compound was carried out.

Fig. 1 shows the asymmetric unit consisting of two molecules of the title compound. The naphthalene ring system is itself planar and the benzofuran ring system is also planar with a maximum deviation of 0.039 (1), 0.008 (1) Å, respectively in one molecule and 0.022 (2), 0.033 (1) Å in the other. The dihedral angles between the naphthalene ring systems and the benzofuran ring systems are 76.49 (7) and 86.17 (7)° in the two molecules. The C7—C8—O1—C11 [179.12 (15)°] and C29—C28—O4—C30 [179.87 (140)°] torsional angles that indicate the methoxy substitutions are essentially coplanar with the attached ring.

Hydrogen bond interactions are shown in Table 1 and the C—H···π interactions (*Cg*2, *Cg*4 and *Cg*8 are centriods of the rings containing the atoms C1—C6, C13—C18 and C20—C25 respectively). In the crystal structure, molecules are linked by intermolecular C—H···O hydrogen-bonding interactions into chains running parallel to the *a* axis.

### Experimental

A mixture of 2-hydrazinopyridine and tolualdehyde were refluxed in ethanol with a catalytic quantity of conc. HCl or gl. AcOH. After the reaction is over, the contents were cooled down and the resulting product was filtered off. Diffraction quality crystals were obtained upon recrystallization in ethanol.

### Refinement

The C bound H atoms positioned geometrically (C—H = 0.93–0.98 Å) and allowed to ride on their parent atoms, with 1.5*U*_eq_(C) for methyl H and 1.2 *U*_eq_(C) for other H atoms.

### Crystal data

**Table:**

C_19_H_14_O_3_	*F*(000) = 1216
*M**_r_* = 290.30	*D*_x_ = 1.319 Mg m^−^^3^
Monoclinic, *P*2_1_/*c*	Mo *K*α radiation, λ = 0.71073 Å
Hall symbol: -P 2ybc	Cell parameters from 1125 reflections
*a* = 13.2572 (5) Å	θ = 1.5–28.3°
*b* = 11.8560 (4) Å	µ = 0.09 mm^−^^1^
*c* = 18.6160 (7) Å	*T* = 293 K
β = 91.657 (2)°	Block, colourless
*V* = 2924.79 (18) Å^3^	0.24 × 0.22 × 0.2 mm
*Z* = 8	

### Data collection

**Table:**

Bruker SMART APEXII area-detector diffractometer	7292 independent reflections
Radiation source: fine-focus sealed tube	4519 reflections with *I* > 2σ(*I*)
graphite	*R*_int_ = 0.027
ω and φ scans	θ_max_ = 28.3°, θ_min_ = 1.5°
Absorption correction: multi-scan (*SADABS*; Bruker, 2008)	*h* = −17→17
*T*_min_ = 0.979, *T*_max_ = 0.982	*k* = −15→12
27565 measured reflections	*l* = −24→21

### Refinement

**Table:**

Refinement on *F*^2^	Primary atom site location: structure-invariant direct methods
Least-squares matrix: full	Secondary atom site location: difference Fourier map
*R*[*F*^2^ > 2σ(*F*^2^)] = 0.043	Hydrogen site location: inferred from neighbouring sites
*wR*(*F*^2^) = 0.122	H-atom parameters constrained
*S* = 1.00	*w* = 1/[σ^2^(*F*_o_^2^) + (0.0514*P*)^2^ + 0.4948*P*] where *P* = (*F*_o_^2^ + 2*F*_c_^2^)/3
7292 reflections	(Δ/σ)_max_ = 0.001
399 parameters	Δρ_max_ = 0.15 e Å^−^^3^
0 restraints	Δρ_min_ = −0.16 e Å^−^^3^

### Special details

**Table:**

Geometry. All e.s.d.'s (except the e.s.d. in the dihedral angle between two l.s. planes) are estimated using the full covariance matrix. The cell e.s.d.'s are taken into account individually in the estimation of e.s.d.'s in distances, angles and torsion angles; correlations between e.s.d.'s in cell parameters are only used when they are defined by crystal symmetry. An approximate (isotropic) treatment of cell e.s.d.'s is used for estimating e.s.d.'s involving l.s. planes.
Refinement. Refinement of *F*^2^ against ALL reflections. The weighted *R*-factor *wR* and goodness of fit *S* are based on *F*^2^, conventional *R*-factors *R* are based on *F*, with *F* set to zero for negative *F*^2^. The threshold expression of *F*^2^ > σ(*F*^2^) is used only for calculating *R*-factors(gt) *etc*. and is not relevant to the choice of reflections for refinement. *R*-factors based on *F*^2^ are statistically about twice as large as those based on *F*, and *R*- factors based on ALL data will be even larger.

### Fractional atomic coordinates and isotropic or equivalent isotropic displacement parameters (Å^2^)

**Table:**

	*x*	*y*	*z*	*U*_iso_*/*U*_eq_	
C1	0.41021 (11)	0.73457 (15)	0.18630 (8)	0.0555 (4)	
H1	0.4191	0.6579	0.1781	0.067*	
C2	0.34451 (13)	0.76890 (18)	0.23690 (9)	0.0676 (5)	
H2	0.3092	0.7151	0.2624	0.081*	
C3	0.32915 (14)	0.88307 (19)	0.25124 (10)	0.0734 (5)	
H3	0.2832	0.9051	0.2854	0.088*	
C4	0.38149 (14)	0.96087 (17)	0.21512 (10)	0.0691 (5)	
H4	0.3718	1.0369	0.2251	0.083*	
C5	0.45096 (11)	0.92985 (14)	0.16230 (8)	0.0518 (4)	
C6	0.46531 (10)	0.81381 (12)	0.14591 (7)	0.0438 (3)	
C7	0.53569 (10)	0.78300 (12)	0.09255 (8)	0.0428 (3)	
C8	0.59224 (10)	0.86679 (13)	0.06143 (8)	0.0477 (4)	
C9	0.57635 (12)	0.98140 (14)	0.07692 (9)	0.0560 (4)	
H9	0.6129	1.0367	0.0536	0.067*	
C10	0.50777 (13)	1.01078 (14)	0.12582 (10)	0.0604 (4)	
H10	0.4978	1.0868	0.1357	0.072*	
C11	0.72638 (15)	0.91029 (19)	−0.01797 (12)	0.0838 (6)	
H11A	0.6843	0.9610	−0.0457	0.126*	
H11B	0.7710	0.8714	−0.0493	0.126*	
H11C	0.7653	0.9523	0.0171	0.126*	
C12	0.55068 (10)	0.66110 (12)	0.07285 (8)	0.0459 (3)	
H12	0.4906	0.6189	0.0865	0.055*	
C13	0.57246 (10)	0.63310 (12)	−0.00365 (8)	0.0450 (3)	
C14	0.51875 (12)	0.65709 (14)	−0.06670 (9)	0.0597 (4)	
H14	0.4573	0.6948	−0.0664	0.072*	
C15	0.56080 (16)	0.62235 (16)	−0.13052 (10)	0.0711 (5)	
H15	0.5269	0.6381	−0.1738	0.085*	
C16	0.65141 (15)	0.56512 (16)	−0.13148 (10)	0.0689 (5)	
H16	0.6777	0.5437	−0.1752	0.083*	
C17	0.70303 (13)	0.53953 (14)	−0.06901 (9)	0.0587 (4)	
H17	0.7635	0.4999	−0.0693	0.070*	
C18	0.66194 (10)	0.57481 (12)	−0.00517 (8)	0.0460 (3)	
C19	0.70285 (11)	0.56471 (13)	0.06827 (9)	0.0514 (4)	
O1	0.66568 (8)	0.83143 (10)	0.01706 (7)	0.0691 (3)	
O2	0.63802 (8)	0.61512 (9)	0.11334 (5)	0.0532 (3)	
O3	0.77989 (9)	0.52232 (11)	0.09071 (7)	0.0748 (4)	
C20	1.22184 (12)	0.35822 (17)	0.29788 (9)	0.0630 (5)	
H20	1.2703	0.3233	0.3274	0.076*	
C21	1.20853 (13)	0.47138 (18)	0.30274 (9)	0.0662 (5)	
H21	1.2475	0.5137	0.3352	0.079*	
C22	1.13564 (13)	0.52358 (15)	0.25850 (9)	0.0599 (4)	
H22	1.1268	0.6013	0.2614	0.072*	
C23	1.07740 (12)	0.46323 (13)	0.21134 (8)	0.0521 (4)	
H23	1.0291	0.5005	0.1829	0.063*	
C24	1.08833 (10)	0.34463 (12)	0.20421 (7)	0.0438 (3)	
C25	1.16370 (11)	0.29228 (14)	0.24895 (8)	0.0498 (4)	
C26	1.17846 (12)	0.17547 (15)	0.24275 (9)	0.0592 (4)	
H26	1.2266	0.1406	0.2725	0.071*	
C27	1.12480 (12)	0.11230 (14)	0.19494 (9)	0.0578 (4)	
H27	1.1372	0.0353	0.1914	0.069*	
C28	1.05014 (11)	0.16302 (13)	0.15056 (8)	0.0479 (4)	
C29	1.03008 (10)	0.27724 (12)	0.15521 (7)	0.0426 (3)	
C30	1.01050 (15)	−0.01427 (14)	0.09473 (10)	0.0672 (5)	
H30A	1.0780	−0.0264	0.0792	0.101*	
H30B	0.9634	−0.0455	0.0600	0.101*	
H30C	1.0021	−0.0504	0.1403	0.101*	
C31	0.94556 (10)	0.32783 (12)	0.10989 (8)	0.0438 (3)	
H31	0.9436	0.4090	0.1196	0.053*	
C32	0.94656 (11)	0.31139 (12)	0.03025 (8)	0.0470 (3)	
C33	1.02020 (14)	0.33418 (15)	−0.01830 (9)	0.0654 (5)	
H33	1.0826	0.3631	−0.0035	0.079*	
C34	0.9977 (2)	0.31242 (18)	−0.08992 (11)	0.0870 (7)	
H34	1.0464	0.3260	−0.1239	0.104*	
C35	0.9044 (2)	0.27095 (18)	−0.11231 (11)	0.0908 (7)	
H35	0.8911	0.2583	−0.1610	0.109*	
C36	0.83122 (17)	0.24824 (15)	−0.06370 (11)	0.0753 (6)	
H36	0.7684	0.2203	−0.0784	0.090*	
C37	0.85471 (12)	0.26858 (13)	0.00780 (9)	0.0529 (4)	
C38	0.79453 (12)	0.24820 (13)	0.07115 (10)	0.0570 (4)	
O4	0.99266 (9)	0.10388 (9)	0.10173 (6)	0.0598 (3)	
O5	0.84930 (7)	0.27923 (9)	0.13030 (6)	0.0530 (3)	
O6	0.71107 (9)	0.20914 (12)	0.07622 (9)	0.0861 (4)	

### Atomic displacement parameters (Å^2^)

**Table:**

	*U*^11^	*U*^22^	*U*^33^	*U*^12^	*U*^13^	*U*^23^
C1	0.0534 (8)	0.0582 (10)	0.0553 (9)	−0.0061 (7)	0.0103 (7)	−0.0071 (8)
C2	0.0605 (10)	0.0846 (14)	0.0585 (11)	−0.0090 (9)	0.0161 (8)	−0.0075 (9)
C3	0.0623 (10)	0.0955 (16)	0.0631 (11)	0.0115 (10)	0.0165 (9)	−0.0168 (11)
C4	0.0679 (11)	0.0690 (12)	0.0706 (12)	0.0218 (9)	0.0060 (9)	−0.0158 (10)
C5	0.0497 (8)	0.0514 (10)	0.0540 (9)	0.0090 (7)	−0.0053 (7)	−0.0058 (7)
C6	0.0383 (7)	0.0485 (9)	0.0446 (8)	0.0014 (6)	−0.0024 (6)	−0.0043 (7)
C7	0.0382 (7)	0.0436 (8)	0.0466 (8)	−0.0014 (6)	0.0014 (6)	−0.0033 (6)
C8	0.0431 (7)	0.0516 (9)	0.0484 (8)	−0.0047 (6)	0.0024 (6)	−0.0012 (7)
C9	0.0593 (9)	0.0454 (10)	0.0630 (10)	−0.0071 (7)	−0.0045 (8)	0.0061 (8)
C10	0.0675 (10)	0.0441 (10)	0.0692 (11)	0.0072 (8)	−0.0053 (9)	−0.0030 (8)
C11	0.0716 (12)	0.0976 (16)	0.0834 (14)	−0.0200 (11)	0.0234 (10)	0.0152 (12)
C12	0.0412 (7)	0.0451 (9)	0.0519 (9)	−0.0031 (6)	0.0086 (6)	−0.0031 (7)
C13	0.0439 (7)	0.0404 (8)	0.0507 (8)	−0.0045 (6)	0.0027 (6)	−0.0043 (7)
C14	0.0568 (9)	0.0568 (11)	0.0648 (11)	−0.0010 (8)	−0.0115 (8)	−0.0046 (8)
C15	0.0952 (14)	0.0686 (12)	0.0487 (10)	−0.0125 (11)	−0.0121 (9)	−0.0042 (9)
C16	0.0913 (13)	0.0648 (12)	0.0513 (10)	−0.0121 (10)	0.0165 (9)	−0.0106 (9)
C17	0.0616 (9)	0.0530 (10)	0.0626 (11)	0.0007 (8)	0.0179 (8)	−0.0075 (8)
C18	0.0473 (7)	0.0413 (8)	0.0496 (8)	−0.0015 (6)	0.0068 (6)	−0.0016 (7)
C19	0.0528 (8)	0.0424 (9)	0.0590 (10)	0.0047 (7)	0.0019 (7)	0.0028 (7)
O1	0.0654 (7)	0.0627 (8)	0.0810 (8)	−0.0175 (6)	0.0313 (6)	−0.0063 (6)
O2	0.0633 (6)	0.0513 (7)	0.0452 (6)	0.0078 (5)	0.0023 (5)	0.0023 (5)
O3	0.0693 (8)	0.0728 (9)	0.0817 (9)	0.0253 (6)	−0.0110 (6)	0.0048 (7)
C20	0.0580 (9)	0.0837 (14)	0.0467 (9)	0.0084 (9)	−0.0081 (7)	0.0004 (9)
C21	0.0659 (10)	0.0841 (14)	0.0485 (10)	−0.0049 (9)	−0.0033 (8)	−0.0093 (9)
C22	0.0696 (10)	0.0568 (11)	0.0535 (10)	−0.0023 (8)	0.0024 (8)	−0.0047 (8)
C23	0.0569 (9)	0.0504 (10)	0.0489 (9)	0.0041 (7)	−0.0015 (7)	0.0031 (7)
C24	0.0441 (7)	0.0480 (9)	0.0395 (8)	0.0051 (6)	0.0071 (6)	0.0056 (6)
C25	0.0473 (8)	0.0619 (11)	0.0404 (8)	0.0101 (7)	0.0036 (6)	0.0051 (7)
C26	0.0572 (9)	0.0678 (12)	0.0524 (9)	0.0226 (8)	−0.0029 (7)	0.0102 (8)
C27	0.0640 (9)	0.0488 (10)	0.0608 (10)	0.0201 (8)	0.0034 (8)	0.0085 (8)
C28	0.0518 (8)	0.0446 (9)	0.0473 (8)	0.0074 (7)	0.0042 (7)	0.0040 (7)
C29	0.0435 (7)	0.0415 (8)	0.0429 (8)	0.0075 (6)	0.0037 (6)	0.0062 (6)
C30	0.0928 (13)	0.0409 (10)	0.0685 (11)	0.0084 (9)	0.0117 (10)	−0.0036 (8)
C31	0.0419 (7)	0.0379 (8)	0.0516 (9)	0.0027 (6)	0.0015 (6)	0.0049 (6)
C32	0.0528 (8)	0.0382 (8)	0.0501 (8)	0.0079 (6)	−0.0001 (7)	0.0091 (7)
C33	0.0729 (11)	0.0626 (12)	0.0615 (11)	0.0085 (9)	0.0119 (9)	0.0202 (9)
C34	0.1293 (19)	0.0723 (14)	0.0608 (13)	0.0321 (13)	0.0279 (13)	0.0245 (10)
C35	0.160 (2)	0.0598 (13)	0.0514 (12)	0.0322 (14)	−0.0157 (14)	0.0026 (10)
C36	0.1050 (15)	0.0499 (11)	0.0690 (13)	0.0135 (10)	−0.0301 (12)	−0.0001 (9)
C37	0.0610 (9)	0.0383 (8)	0.0587 (10)	0.0085 (7)	−0.0127 (8)	0.0035 (7)
C38	0.0469 (8)	0.0427 (9)	0.0810 (12)	0.0037 (7)	−0.0056 (8)	0.0076 (8)
O4	0.0742 (7)	0.0388 (6)	0.0659 (7)	0.0071 (5)	−0.0074 (6)	−0.0018 (5)
O5	0.0443 (5)	0.0545 (7)	0.0605 (7)	0.0050 (5)	0.0063 (5)	0.0078 (5)
O6	0.0511 (7)	0.0750 (9)	0.1318 (13)	−0.0123 (6)	−0.0054 (7)	0.0109 (8)

### Geometric parameters (Å, °)

**Table:**

C1—C2	1.363 (2)	C20—C21	1.356 (3)
C1—C6	1.419 (2)	C20—C25	1.412 (2)
C1—H1	0.9300	C20—H20	0.9300
C2—C3	1.396 (3)	C21—C22	1.396 (2)
C2—H2	0.9300	C21—H21	0.9300
C3—C4	1.346 (3)	C22—C23	1.356 (2)
C3—H3	0.9300	C22—H22	0.9300
C4—C5	1.415 (2)	C23—C24	1.420 (2)
C4—H4	0.9300	C23—H23	0.9300
C5—C10	1.407 (2)	C24—C29	1.423 (2)
C5—C6	1.423 (2)	C24—C25	1.4245 (19)
C6—C7	1.4298 (19)	C25—C26	1.404 (2)
C7—C8	1.382 (2)	C26—C27	1.350 (2)
C7—C12	1.506 (2)	C26—H26	0.9300
C8—O1	1.3611 (18)	C27—C28	1.406 (2)
C8—C9	1.406 (2)	C27—H27	0.9300
C9—C10	1.351 (2)	C28—O4	1.3633 (18)
C9—H9	0.9300	C28—C29	1.383 (2)
C10—H10	0.9300	C29—C31	1.5075 (19)
C11—O1	1.406 (2)	C30—O4	1.4272 (18)
C11—H11A	0.9600	C30—H30A	0.9600
C11—H11B	0.9600	C30—H30B	0.9600
C11—H11C	0.9600	C30—H30C	0.9600
C12—O2	1.4681 (17)	C31—O5	1.4604 (16)
C12—C13	1.499 (2)	C31—C32	1.496 (2)
C12—H12	0.9800	C31—H31	0.9800
C13—C18	1.374 (2)	C32—C37	1.373 (2)
C13—C14	1.385 (2)	C32—C33	1.376 (2)
C14—C15	1.389 (2)	C33—C34	1.382 (3)
C14—H14	0.9300	C33—H33	0.9300
C15—C16	1.380 (3)	C34—C35	1.384 (3)
C15—H15	0.9300	C34—H34	0.9300
C16—C17	1.366 (3)	C35—C36	1.372 (3)
C16—H16	0.9300	C35—H35	0.9300
C17—C18	1.386 (2)	C36—C37	1.379 (2)
C17—H17	0.9300	C36—H36	0.9300
C18—C19	1.461 (2)	C37—C38	1.463 (2)
C19—O3	1.2023 (18)	C38—O6	1.2057 (19)
C19—O2	1.3570 (18)	C38—O5	1.352 (2)
			
C2—C1—C6	121.16 (16)	C21—C20—C25	121.40 (15)
C2—C1—H1	119.4	C21—C20—H20	119.3
C6—C1—H1	119.4	C25—C20—H20	119.3
C1—C2—C3	121.42 (18)	C20—C21—C22	119.22 (16)
C1—C2—H2	119.3	C20—C21—H21	120.4
C3—C2—H2	119.3	C22—C21—H21	120.4
C4—C3—C2	119.27 (17)	C23—C22—C21	121.29 (17)
C4—C3—H3	120.4	C23—C22—H22	119.4
C2—C3—H3	120.4	C21—C22—H22	119.4
C3—C4—C5	121.62 (17)	C22—C23—C24	121.67 (15)
C3—C4—H4	119.2	C22—C23—H23	119.2
C5—C4—H4	119.2	C24—C23—H23	119.2
C10—C5—C4	121.77 (16)	C23—C24—C29	124.11 (13)
C10—C5—C6	118.63 (14)	C23—C24—C25	116.65 (13)
C4—C5—C6	119.60 (16)	C29—C24—C25	119.24 (13)
C1—C6—C5	116.90 (13)	C26—C25—C20	121.63 (14)
C1—C6—C7	123.73 (14)	C26—C25—C24	118.61 (14)
C5—C6—C7	119.33 (13)	C20—C25—C24	119.76 (15)
C8—C7—C6	118.71 (13)	C27—C26—C25	121.96 (14)
C8—C7—C12	120.73 (13)	C27—C26—H26	119.0
C6—C7—C12	120.51 (12)	C25—C26—H26	119.0
O1—C8—C7	116.06 (14)	C26—C27—C28	119.92 (15)
O1—C8—C9	122.42 (14)	C26—C27—H27	120.0
C7—C8—C9	121.50 (14)	C28—C27—H27	120.0
C10—C9—C8	119.68 (15)	O4—C28—C29	116.14 (12)
C10—C9—H9	120.2	O4—C28—C27	122.84 (14)
C8—C9—H9	120.2	C29—C28—C27	121.02 (15)
C9—C10—C5	121.98 (16)	C28—C29—C24	119.20 (12)
C9—C10—H10	119.0	C28—C29—C31	119.71 (13)
C5—C10—H10	119.0	C24—C29—C31	121.06 (12)
O1—C11—H11A	109.5	O4—C30—H30A	109.5
O1—C11—H11B	109.5	O4—C30—H30B	109.5
H11A—C11—H11B	109.5	H30A—C30—H30B	109.5
O1—C11—H11C	109.5	O4—C30—H30C	109.5
H11A—C11—H11C	109.5	H30A—C30—H30C	109.5
H11B—C11—H11C	109.5	H30B—C30—H30C	109.5
O2—C12—C13	103.58 (11)	O5—C31—C32	103.85 (11)
O2—C12—C7	109.82 (11)	O5—C31—C29	109.78 (11)
C13—C12—C7	118.33 (12)	C32—C31—C29	118.36 (12)
O2—C12—H12	108.2	O5—C31—H31	108.1
C13—C12—H12	108.2	C32—C31—H31	108.1
C7—C12—H12	108.2	C29—C31—H31	108.1
C18—C13—C14	120.59 (14)	C37—C32—C33	120.82 (15)
C18—C13—C12	108.74 (13)	C37—C32—C31	108.46 (13)
C14—C13—C12	130.67 (14)	C33—C32—C31	130.70 (15)
C13—C14—C15	117.05 (16)	C32—C33—C34	117.35 (19)
C13—C14—H14	121.5	C32—C33—H33	121.3
C15—C14—H14	121.5	C34—C33—H33	121.3
C16—C15—C14	121.85 (17)	C33—C34—C35	121.5 (2)
C16—C15—H15	119.1	C33—C34—H34	119.2
C14—C15—H15	119.1	C35—C34—H34	119.2
C17—C16—C15	120.85 (16)	C36—C35—C34	120.91 (19)
C17—C16—H16	119.6	C36—C35—H35	119.5
C15—C16—H16	119.6	C34—C35—H35	119.5
C16—C17—C18	117.59 (16)	C35—C36—C37	117.3 (2)
C16—C17—H17	121.2	C35—C36—H36	121.4
C18—C17—H17	121.2	C37—C36—H36	121.4
C13—C18—C17	122.04 (15)	C32—C37—C36	122.13 (18)
C13—C18—C19	108.61 (13)	C32—C37—C38	108.36 (14)
C17—C18—C19	129.28 (14)	C36—C37—C38	129.49 (17)
O3—C19—O2	121.07 (15)	O6—C38—O5	120.88 (17)
O3—C19—C18	130.39 (15)	O6—C38—C37	130.50 (17)
O2—C19—C18	108.53 (12)	O5—C38—C37	108.61 (13)
C8—O1—C11	120.37 (14)	C28—O4—C30	118.35 (13)
C19—O2—C12	110.51 (11)	C38—O5—C31	110.37 (12)
			
C6—C1—C2—C3	−0.3 (3)	C25—C20—C21—C22	0.1 (3)
C1—C2—C3—C4	−1.0 (3)	C20—C21—C22—C23	0.6 (3)
C2—C3—C4—C5	0.7 (3)	C21—C22—C23—C24	−0.6 (2)
C3—C4—C5—C10	−178.27 (17)	C22—C23—C24—C29	−179.37 (14)
C3—C4—C5—C6	0.9 (3)	C22—C23—C24—C25	−0.2 (2)
C2—C1—C6—C5	1.9 (2)	C21—C20—C25—C26	178.79 (16)
C2—C1—C6—C7	179.56 (15)	C21—C20—C25—C24	−0.9 (2)
C10—C5—C6—C1	177.05 (14)	C23—C24—C25—C26	−178.81 (14)
C4—C5—C6—C1	−2.1 (2)	C29—C24—C25—C26	0.4 (2)
C10—C5—C6—C7	−0.8 (2)	C23—C24—C25—C20	0.9 (2)
C4—C5—C6—C7	−179.93 (14)	C29—C24—C25—C20	−179.88 (13)
C1—C6—C7—C8	−173.84 (13)	C20—C25—C26—C27	−178.43 (16)
C5—C6—C7—C8	3.8 (2)	C24—C25—C26—C27	1.2 (2)
C1—C6—C7—C12	3.8 (2)	C25—C26—C27—C28	−1.3 (3)
C5—C6—C7—C12	−178.57 (12)	C26—C27—C28—O4	−179.32 (15)
C6—C7—C8—O1	172.94 (12)	C26—C27—C28—C29	−0.3 (2)
C12—C7—C8—O1	−4.7 (2)	O4—C28—C29—C24	−178.96 (12)
C6—C7—C8—C9	−5.1 (2)	C27—C28—C29—C24	2.0 (2)
C12—C7—C8—C9	177.31 (13)	O4—C28—C29—C31	2.57 (19)
O1—C8—C9—C10	−174.67 (15)	C27—C28—C29—C31	−176.51 (13)
C7—C8—C9—C10	3.2 (2)	C23—C24—C29—C28	177.19 (14)
C8—C9—C10—C5	0.0 (2)	C25—C24—C29—C28	−2.0 (2)
C4—C5—C10—C9	177.98 (16)	C23—C24—C29—C31	−4.4 (2)
C6—C5—C10—C9	−1.2 (2)	C25—C24—C29—C31	176.45 (12)
C8—C7—C12—O2	81.43 (16)	C28—C29—C31—O5	63.33 (17)
C6—C7—C12—O2	−96.15 (14)	C24—C29—C31—O5	−115.11 (14)
C8—C7—C12—C13	−37.12 (19)	C28—C29—C31—C32	−55.56 (18)
C6—C7—C12—C13	145.30 (13)	C24—C29—C31—C32	126.00 (14)
O2—C12—C13—C18	1.35 (15)	O5—C31—C32—C37	5.82 (15)
C7—C12—C13—C18	123.12 (14)	C29—C31—C32—C37	127.76 (14)
O2—C12—C13—C14	−178.30 (15)	O5—C31—C32—C33	−175.73 (15)
C7—C12—C13—C14	−56.5 (2)	C29—C31—C32—C33	−53.8 (2)
C18—C13—C14—C15	−1.7 (2)	C37—C32—C33—C34	0.1 (2)
C12—C13—C14—C15	177.97 (15)	C31—C32—C33—C34	−178.20 (16)
C13—C14—C15—C16	0.8 (3)	C32—C33—C34—C35	0.9 (3)
C14—C15—C16—C17	0.6 (3)	C33—C34—C35—C36	−1.0 (3)
C15—C16—C17—C18	−1.1 (3)	C34—C35—C36—C37	0.1 (3)
C14—C13—C18—C17	1.2 (2)	C33—C32—C37—C36	−1.1 (2)
C12—C13—C18—C17	−178.54 (14)	C31—C32—C37—C36	177.56 (14)
C14—C13—C18—C19	178.39 (14)	C33—C32—C37—C38	177.33 (14)
C12—C13—C18—C19	−1.31 (16)	C31—C32—C37—C38	−4.03 (16)
C16—C17—C18—C13	0.3 (2)	C35—C36—C37—C32	1.0 (2)
C16—C17—C18—C19	−176.36 (16)	C35—C36—C37—C38	−177.07 (17)
C13—C18—C19—O3	−178.41 (17)	C32—C37—C38—O6	−178.17 (17)
C17—C18—C19—O3	−1.4 (3)	C36—C37—C38—O6	0.1 (3)
C13—C18—C19—O2	0.74 (17)	C32—C37—C38—O5	0.50 (17)
C17—C18—C19—O2	177.71 (15)	C36—C37—C38—O5	178.75 (15)
C7—C8—O1—C11	179.12 (15)	C29—C28—O4—C30	179.87 (14)
C9—C8—O1—C11	−2.9 (2)	C27—C28—O4—C30	−1.1 (2)
O3—C19—O2—C12	179.41 (14)	O6—C38—O5—C31	−177.81 (14)
C18—C19—O2—C12	0.16 (16)	C37—C38—O5—C31	3.38 (16)
C13—C12—O2—C19	−0.90 (15)	C32—C31—O5—C38	−5.60 (14)
C7—C12—O2—C19	−128.20 (12)	C29—C31—O5—C38	−133.08 (12)

### Hydrogen-bond geometry (Å, °)

**Table:**

*Cg*2, *Cg*4 and *Cg*8 are the centroids of C1–C6, C13–C18 and C20–C25 rings, respectively.

**Table:**

*D*—H···*A*	*D*—H	H···*A*	*D*···*A*	*D*—H···*A*
C9—H9···O6^i^	0.93	2.45	3.238 (2)	142
C14—H14···O6^ii^	0.93	2.51	3.435 (2)	175
C12—H12···Cg4^ii^	0.98	2.93	3.755 (2)	143
C15—H15···Cg2^iii^	0.93	2.88	3.800 (2)	169
C16—H16···Cg8^iv^	0.93	2.81	3.553 (2)	137
C30—H30C···Cg8^v^	0.96	2.89	3.687 (2)	141

Symmetry codes: (i) *x*, *y*+1, *z*; (ii) −*x*+1, −*y*+1, −*z*; (iii) *x*, −*y*+1/2, *z*−3/2; (iv) −*x*+2, −*y*+1, −*z*; (v) −*x*+2, *y*−1/2, −*z*+1/2.
